# Recurrent Malarial Attacks

**Published:** 1882-01-20

**Authors:** R. L. Hinton

**Affiliations:** Prescott, Arkansas


### RECURRENT MALARIAL ATTACKS.

*Editors of The Southern Medical Record—*

GENTLEMEN: In the November number of the RECORD, I notice an article by Dr. John H. Pool, of South Mills, North Carolina, taken from the Virginia Medical Monthly, entitled "Recurrent Obstinate Malarial Attacks," commonly called in this section "Chronic Chills." I concur fully with the doctor in all he says in regard to regular periodic return of the paroxysms, and also his plan of meeting them with quinine, and I used to think well of his alterative treatment, of Lugol's solution, etc., until I found a better in the hyposulphite of sodium, which I give after each meal (in such dose as I think the case will bear, or require) in solution, with the extract of licorice to cover the taste, or with some aromatic to suit the whim of the patient.

I apply about twice a week, over the spleen and liver, the tincture of iodine, say enough to burn slightly. I have never found any other treatment equal to this, in softening and melting down these enlarged and indurated livers and spleens, and thus making a radical or final cure of this troublesome disease. I have suggested this treatment to other physicians, who can say the same of their experience.

If you think the above of interest sufficient to publish, or any part of it, you are at liberty to use it as you see fit.

Respectfully, &c., R. L. HINTON, M. D.

*Prescott, Arkansas, December,* 1881.

